# Trigeminal neurons control immune-bone cell interaction and metabolism in apical periodontitis

**DOI:** 10.1007/s00018-022-04335-w

**Published:** 2022-05-31

**Authors:** Obadah N. Austah, Katherine V. Lillis, Armen N. Akopian, Stephen E. Harris, Ruta Grinceviciute, Anibal Diogenes

**Affiliations:** 1 Department of Endodontics, University of Texas Health Science Center at San Antonio, 7703 Floyd Curl Dr, San Antonio, TX 78229 USA; 2 Department of Endodontics, Faculty of Dentistry, King Abdulaziz University, Jeddah, Saudi Arabia; 3 Department of Periodontics, University of Texas Health Science Center at San Antonio, San Antonio, TX, USA

**Keywords:** Sensory, Neurons, Nociceptor, Bone, Nav1.8, Osteolytic

## Abstract

**Abstract:**

Apical periodontitis (AP) is an inflammatory disease occurring following tooth infection with distinct osteolytic activity. Despite increasing evidence that sensory neurons participate in regulation of non-neuronal cells, their role in the development of AP is largely unknown. We hypothesized that trigeminal ganglia (TG) Nav1.8^+^ nociceptors regulate bone metabolism changes in response to AP. A selective ablation of nociceptive neurons in Nav1.8^Cre^/Diphtheria toxin A (DTA)^Lox^ mouse line was used to evaluate the development and progression of AP using murine model of infection-induced AP. Ablation of Nav1.8^+^ nociceptors had earlier progression of AP with larger osteolytic lesions. Immunohistochemical and RNAscope analyses demonstrated greater number of macrophages, T-cells, osteoclast and osteoblast precursors and an increased RANKL:OPG ratio at earlier time points among Nav1.8^Cre^/ DTA^Lox^ mice. There was an increased expression of IL-1α and IL-6 within lesions of nociceptor-ablated mice. Further, co-culture experiments demonstrated that TG neurons promoted osteoblast mineralization and inhibited osteoclastic function. The findings suggest that TG Nav1.8^+^ neurons contribute to modulation of the AP development by delaying the influx of immune cells, promoting osteoblastic differentiation, and decreasing osteoclastic activities. This newly uncovered mechanism could become a therapeutic strategy for the treatment of AP and minimize the persistence of osteolytic lesions in refractory cases.

**Graphical abstract:**

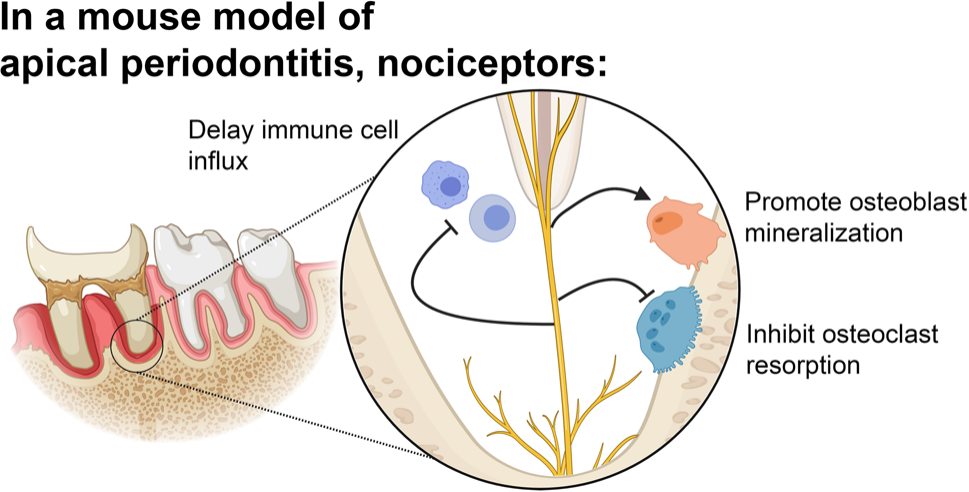

**Supplementary Information:**

The online version contains supplementary material available at 10.1007/s00018-022-04335-w.

## Introduction

Dental infections are a prevalent global health problem resulting in significant human suffering, premature tooth loss, and decrease in quality of life and productivity [1]. Ingress of microorganisms, that are normally occurring in the oral cavity, into the mineralized dental structures is marked by robust inflammation and severe pain [2]. As the disease progresses, the inflammation extends apically resulting in the development of apical periodontitis (AP) that is often accompanied by substantial bone remodeling [3]. This change in bone development and differentiation is believed to result from a shift between osteoblastic and osteoclastic activity, favoring the loss of mineralized bone matrix. Some of the complex immunologic events leading to this bone metabolism imbalance include the diffusion of microbial antigens through the apical opening of the root canals with the subsequent activation of cells characteristic for the innate and adaptive immune response [4]. Although the role of immune cells and key cytokines in bone remodeling due to dental infections has been previously investigated [5, 6], far less is known for the role of trigeminal afferent neurons, which are abundantly present in relevant tissues, in this pathologic process.

There is an increasing body of evidence that peripheral sensory neurons participate in skeletal homeostasis through elaborate cross-talk with other non-neuronal cells in the appendicular skeleton [7]. Thus, it is agreed that craniodental homeostasis is closely influenced by nociceptors, particularly how these pain-sensing fibers participate in the dysregulation of bone metabolism following dental infections [8, 9]. Dental structures are densely innervated with nociceptors, and we have demonstrated that the pharmacological ablation of capsaicin-sensitive neurons led to an early onset of bone loss following dental infections in rats [8]. Despite this novel observation, the mechanism by which peripheral sensory neurons regulate this increased bone loss following dental infection is still largely unknown.

The pharmacological ablation by capsaicin affects neurons expressing the transient receptor vanilloid receptor-1 (TRPV1) only affects a subpopulation of nociceptors [10]. It was demonstrated that a third of the dental innervating neurons are TRPV1^+^, while as much as 60% dental nerve fibers contain calcitonin gene-related peptide (CGRP) [11]. Additionally, TRPV1 has been found on multiple cell types including immune cells, which makes the pharmacological intervention less likely to be merely neuronal-selective [12]. This indicates that other tools affecting broader subset of sensory neurons need to be applied for through investigation of bone metabolism regulation by the trigeminal ganglia (TG) sensory neurons. In this respect, TG neurons could be divided into unmyelinated C-fibers, lightly myelinated A-delta fibers (A-δ), and heavily myelinated A-beta (A-β) fibers [13]. Recent studies have further classified sensory neurons using advanced molecular profiling and transgenic tools into various distinct subsets [14–16]. Close comparative analysis showed that a hallmark of nociceptors is expression of the tetrodotoxin-resistant (TTX-R) voltage gated sodium channel (Nav1.8), thus making it a suitable molecular marker for manipulating broad spectrum of nociceptors [17]. The use of Cre-loxP system has been efficiently utilized to generate mouse lines with either conditional knockout or reporter mice for specific marker-genes [15, 18]. This genetic tool can be applied to ablate defined cell types. Thus, the crossing of Nav1.8^Cre^ and diphtheria toxin (DTA)^Lox^ mice can lead to double heterozygous offspring in which Nav1.8-positive cells are ablated through DTA expression-induced toxicity. We have utilized this approach to isolate and study roles of Nav1.8^+^ nociceptors in apical periodontitis bone loss.

Bone metabolism is a dynamic process and mediated by balanced activities of osteoblasts/osteoclasts cells to maintain the bone mass. In inflammatory pathological conditions such as AP, a shift occurs in the balance leading to abnormal osteoclastic or osteoblastic functions, resulting in bone loss [19]. Bone is innervated by sensory nerves, and there is growing evidence that dorsal root ganglia (DRG) sensory neurons participate in skeletal bone homeostasis and remodeling [20, 21]. However, it is known that DRG and TG neurons are cellularly and functionally distinct [14] and the orofacial region is innervated by the TG neurons compared to the rest of the body which is innervated by DRG neurons. In addition, AP resulting from dental infections has a unique pathophysiology with the infected tooth serving as a revoir of a complex microorganism flora whose antigens diffuse through the root foramen resulting in a long-standing robust immunological challenge leading to pain and localized bone loss. Further, the density of periapical innervation has been shown to increase within tissues in AP as result of neuronal sprouting [22], yet the role of sensory innervation in the bone homeostasis of this prevalent disease is largely unknown. Therefore, the aims of the present study were to: (1) to investigate the role of nociceptors (Nav1.8^+^ neurons) in the development of AP using transgenic selective nociceptor ablation and a well-established murine model of infection-induced AP, and (2) to evaluate TG nociceptors regulation of osteoblastic and osteoclastic function using an in vitro co-culture system.

## Methods

### Animals

Animals were housed 2–5 per cage and maintained in a light/temperature control environment with food and water available *ad lib*. Breeding was performed in our facility, and all experiments were performed on 8–12-week-old mice with equal distribution of male and females.

For the Nav1.8^+^ neuronal ablation, Nav1.8^cre+^ mouse line on C57BL/6 J background was provided by the laboratory of John Wood (University College London, UK) [18]. Rosa26^tm1(DTA)Lky+^ on B6.129P2 background were obtained from the Jackson Laboratory (stock# 009669). Neither Nav1.8^cre+^ nor Rosa26^tm1(DTA)Lky+^ mice, which will be used as controls, showed any systemic anomalies and had normal nociceptive responses, as shown previously [18]. Heterozygous Nav1.8^cre+^ mice were crossed with homozygous Rosa26^tm1(DTA)Lky+^ to generate control (Nav1.8 ^cre±^) and Nav1.8^+^ neurons (nociceptors) ablated mice using Nav1.8 ^cre±^ DTA^lox±^ (Nav1.8-DTA). Genomic DNA was isolated from tail snips using the REDExtract-N-Amp Tissue PCR Kit (Sigma) and genotyping performed by Polymerase chain reaction (PCR) with primers specific for Nav1.8 and DTA (see supplemental materials). Behavioral testing was performed for both Nav1.8-Cre and Nav1.8-DTA mice using the radiant heat paw withdrawal assay (Hargreaves’s test) [23] and the capsaicin eye-wipe nocifensive behavior test [24].

### Apical periodontitis model

All experiments were performed on 8–10-week-old animals with a sample size of eight mice per group per timepoint with equal distribution of female and male mice. In brief, animals were anesthetized via intraperitoneal (IP) injection of ketamine (75 mg/kg)/dexdomitor (1 mg/kg), as described previously [8]. Animals were mounted on a custom jaw retraction apparatus, and two standardized cavities (i.e., mesial and distal of the pulp chamber) were created on the occlusal surface of the mandibular left molar using a sterile high-speed size #1/4 round bur to the depth of the diameter of the bur with the aid of a surgical microscope (Zeiss). The exposure of the dental pulp to the oral environment was confirmed visually with the insertion of an Endodontic K-file #8 (Dentsply Maillefer) into the mesial and distal canals of the tooth. Next, animals received an intraperitoneal injection of Antisedan (1 mg/kg) to reverse the anesthesia, and they were then housed in a climate-controlled environment with free access to water and food until tissue collection.

### Tissue collection

At the end of each timepoint (3, 7 or 14 days), animals were briefly anesthetized by isoflurane inhalation (for 60–90 s) and sacrificed by cervical dislocation. The mandibles were dissected, fixed for 2 h in 4% paraformaldehyde in 0.1 M phosphate buffer (PB), rinsed and stored in 0.1 M PB (ThermoFisher Scientific) until mandibles were scanned with micro-computed tomography (μCT) and later subjected to immunohistochemistry or RNAscope. Next, dissected surrounding bone and teeth were pooled from three mice per sample, and total RNA was isolated following immediate snap freezing in liquid nitrogen, grinding and homogenization in RNA lysis buffer. In addition, trigeminal ganglia were dissected from the animals at different time points and frozen in RNA*later* (Thermo Fisher Scientific) at -20 °C until use.

### Total RNA isolation and RT-PCR

Total RNA (0.1–0.5 µg) was isolated from harvested periapical lesions and trigeminal ganglia tissues (*n* = 6/group/time point). In addition, total RNA (1 µg) was also isolated from IDG-SW3 co-cultured with primary TG neurons (see below) using the RNAeasy Kit (Qiagen) according to the manufacturer’s recommendation followed by cDNA synthesis using the High-Capacity RNA-to-cDNA kit (Thermo Fisher Scientific).

Real time PCR reactions were conducted using the Taqman™ Fast Advanced Master Mix (Thermo Fisher Scientific) and Taqman™ gene expression assays for in vivo samples and co-culture experiments (see table in supplemental materials) on an ABI 7500 Fast Real-Time PCR System (Thermo Fisher Scientific). The relative expression of each target gene messenger RNA (mRNA) was determined using the comparative delta-delta cycle threshold method (ΔΔCt), after normalization to GAPDH, using the control group as the calibrator as previously reported [25].

### Micro-computed tomography

Mandible samples were scanned in a Micro-Computed Tomography Bruker Skyscan1172 (μCT) system (Bruker) to quantify the volume of the osteolytic lesion in the apical area (i.e., progression of AP). Samples were scanned with the following settings: 60 kV, 167 mA, 0.7° rotation step, four frames averaging, and 8 μm voxel size. The region of interest (ROI) included the osteolytic space surrounding the roots and the periodontal ligament space surrounding both roots of the first mandibular molar as a measure of apical lesion volume. The ROI started at the first sagittal slice, where the mesial full root canal space is visible through the apex and included three slices in both buccal and lingual directions with 50 μm interval. This area was manually outlined by blinded observers using CT-Analyser software (Bruker), and an automated thresholding script was used to measure the total volume of the void space. The mean lesion volume of all slices was normalized to the volume of the periodontal space of the contralateral healthy tooth within subjects. Thus, the intact first molar in the contralateral side for every animal was used as its own control to normalize the acquired data presented as percent of the contralateral healthy tissue.

### Immunohistochemistry (IHC)

Sectioned mandible samples were processed for IHC with corresponding primary antibodies to evaluate the presence of CGRP + neuronal fibers and to quantify numbers of cluster of differentiation 68 (CD68) + macrophages, cluster of differentiation 3 (CD3) + T-cells, osteocalcin + osteoblasts, and Ki67 + progenitor cells, as well as the expression pattern of RANKL and OPG within apical lesions. In brief, fixed mandible samples were decalcified in a 4% EDTA solution at 4 °C for 3–4 weeks until ready for sectioning. Samples were cryo-protected with 30% sucrose in phosphate buffer overnight before they were embedded in Neg 50 (Richard Allan Scientific). Next, samples were serially sectioned at 20 μm in the longitudinal (Sagittal) plane with a cryostat, and tissue sections were collected onto Superfrost glass slides (Fisher Scientific) and stored at − 20 °C until use.

Samples were permeabilized and blocked using Maxpack Immunostaining Media Kit (Active Motif) and incubated with primary antibodies (see supplemental information) overnight, followed by species-appropriate Alexa Fluor IgG secondary antibodies (Thermofisher; Alexa Fluor 488/568 at a 1:200 concentration). Control sections were processed similarly but without primary antibodies. Next, immune reactivity was detected with a Nikon Eclipse 90i equipped with a C1si laser system confocal microscope (Nikon Instruments). Single images were acquired at 10 × and 20 × magnifications with NIS-elements software (Nikon Instruments) with standardized settings by blinded observers. A minimum of three sections from three independent animals (*n* = 3) were used for all IHC analyses. Positively stained cells for each target within the periapical lesion were counted using NIH ImageJ software. Cell counts in the periapical lesion were normalized to the contralateral intact healthy tooth. Lastly, sectioned samples were also stained with hematoxylin and eosin (Dako) following basic histological staining protocol.

### RNAscope^®^

After each timepoint, mandibles were dissected and fixed in 10% Neutral Buffered Formalin (Sigma) for 24 h. The samples were then washed in PBS and placed in ACD Bone Decalcification Buffer (Advanced Cell Diagnostics) at 4ºC for 2 weeks, changing the solution every 2 days. Mandibles were paraffin-embedded, sectioned at 4 μm, and mounted on Superfrost glass slides (Fisher Scientific). The samples were then processed with the RNAscope^®^ 2.5 HD Duplex Detection Kit (Advanced Cell Diagnostics) following manufacturer’s instructions.

A total of three slides per condition (AP or contralateral control) from different samples at each time point was used for each combination of probe pairs. Brightfield images of the periapical lesion were captured at 40 × magnification using a Nikon Ts2 inverted microscope (Nikon Instruments) using NIS-elements software (Nikon Instruments). Relative gene expression within the apical osteolytic lesions was quantified using NIH ImageJ software after color thresholding. Data are presented as area of each of red and green signals over total region of interest.

### Primary trigeminal ganglia neuronal cultures

Trigeminal ganglia were harvested from Nav1.8-DTA and control mice, and neurons were isolated via enzymatic digestion process, as previously described [26, 27]. Single cell neuronal suspensions at the concentration of (~ 5 × 10^4^ neurons/insert) were seeded on 24-well plate polyethylene cell culture inserts (1 μm-pore sizes) (Millipore-Sigma) precoated with 50 μg/ml laminin (Sigma) in growth medium containing Alpha Modified Eagle’s Medium (αMEM) (Life Technologies), 10% heat-inactivated fetal bovine serum (HI-FBS) (Life Technologies), and 1X Glutamine-Penicillin- Streptomycin (Invitrogen). Neurons were allowed to attach for 24 h in 5% CO_2_ and 37 °C in the presence of 10 μM cytosine β-D- arabinofuranoside (Ara-C) (Sigma) to halt the non- neuronal cell proliferation [28, 29]. The inserts containing the neuronal primary cultures were transferred to 24-well plates containing osteoblast precursor cells or induced RAW 264.7 cell lines for osteoblast and osteoclast co-cultures, respectively.

### Osteoblast co-cultures

The osteoblast cell line IDG-SW3 with green fluorescent protein (GFP) expression under the Dentin Matrix Acidic Phosphoprotein 1(DMP1) promoter (Kerafast) or murine calvaria osteoblast precursor cells (MC3T3-E1, subclone 4) (American Type Culture Collection) were cultured at the concentration of (~ 1 × 10^5^/well) in collagen type-1 coated 24-well plated in the same medium of TG neurons but with the addition of ascorbic acid (50 μg/ml) and β-glycerolphosphate (4 mM). Once cells reached 80% confluency, inserts of TG neuronal cultures were transferred to the plates and incubated for 7 or 14 days at 37 °C and 5% CO_2_ with media replaced every 2 days and new neuronal inserts every 7 days.

### RNA sequencing

Total RNA was isolated from IDG-SW3 cells after 14 days culture in the presence of differentiation media, in the presence or absence of cultured TG neurons from either control or Nav1.8-DTA mice (*n* = 2 wells/condition) using the RNAeasy Kit (Qiagen) following the manufacturer’s recommendation. The total RNA was submitted to the Genome Sequencing Facility at UT Health San Antonio for sequencing and analysis [30]. Briefly, samples were sequenced by 100-bp pair-end module, and Fastq sequences were aligned by tophat2 with ucsc-mm10 genome. Then, gene readCounts were counted by HTSeq, and Reads Per Kilobase of transcript, per Million mapped reads (RPKM) were calculated and corrected by qRNA script. Finally, pairwise comparisons were done by DESeq, and differentially expressed genes (DEGs) for further analysis were selected according to following criteria: RPKM > 1, fold change (FC) ≥ 1.5 and adj *p* < 0.05 calculated by Benjamin-Hodjberg test. Gene ontology biological process analyses of DEGs were performed using Protein Analysis Through Evolutionary Relationships (PANTHER) Overrepresentation Test (PANTHER 16.0 Released 20210224) [31]. DEGs were compared against all genes in the *Mus musculus* reference list using a Fisher test and Bonferroni correction. PANTHER software was used to determine enriched biological processes from GO Ontology database (https://doi.org/10.5281/zenodo.5080993 Released 2021-07-02) and provided outputs including Fold Enrichment scores, number of genes present in the annotation data category, and p values [32].

### Osteoblast differentiation and mineralization assays

At the end of the co-culture period of IDG-SW3 with TG cultures (14 days) from either Nav1.8-Cre or nociceptor ablated mice (*n* = 6 wells/group), the percentage of DMP1-expressing cells was quantified following the detection of GFP-positive cells using an EVOS cell imaging system microscope (Life Technologies) at 20X, following nuclear staining with NucBlue™ Live ReadyProbes™ (Life Technologies).

After 7 days co-culture of MC3T3 cells with TG neurons, formation of hydroxyapatite was detected using the Osteoimage™ mineralization assay (Lonza Biosciences) following the manufacturer recommendations. In addition, calcium accumulation was detected and quantified by Alizarin Red staining using the osteogenic mineralization assay (Millipore-Sigma). Further, alkaline phosphatase activity was measured using the fluorometric alkaline phosphatase assay kit (Abcam) following manufacturer’s recommendations. Lastly, conditioned media were collected on the final day of culture, and secreted osteocalcin was quantified using an osteocalcin enzyme-linked immunosorbent assay (ELISA) (MyBioSource). For all experiments, cell viability relative quantification was determined using OZBlue Cell Viability Kit (OZ Biosciences Inc). Fluorescence (Osteoimage and Alkaline Phosphatase (ALP) assays) and absorbance (Alizarin Red Staining and ELISA) of the samples was detected and quantified in a FlexStation 3 Benchtop Multimode Microplate Reader (Molecular Devices). All experiments included 6–8 samples from at least 3 separate experiments. In addition, representative 20 × magnification images were acquired by brightfield and epifluorescence (488 nm wavelength) microscopy at 20 × magnification using an inverted microscope (EVOS cell imaging system).

### In vitro osteoclastogenesis co-culture assay

A bone resorption fluorescent assay kit (Cosmo Bio) was used according to manufacturer’s recommendations. Murine osteoclast precursor RAW264.7 cells were plated in the lower chamber of 24-well plates coated with fluorescently labelled-calcium phosphate at the density of 1 × 10^4^ cells/well in growth medium containing αMEM (Life Technologies), 10% heat inactivated-FBS (Life Technologies), and 1X Glutamine-Penicillin–Streptomycin (Invitrogen) and were allowed to attach for 24 h in 5% CO_2_ and 37ºC. Next, Receptor activator of NF-κB (RANK) ligand (RANKL; 100 ng/ml) (Sigma) and liposaccharides (LPS) from *Escherichia coli* (100 ng/ml) (InvivoGen, CA) were added to the cells to stimulate osteoclast differentiation [33, 34]. Cultured primary TG neurons in inserts were transferred to 24-well plates containing the induced RAW 264.7 cells and co-cultured for 5 days at 37 °C and 5% CO_2_ (*n* = 7 wells/group). In addition, RAW264.7 in the presence or absence of RANKL and LPS were cultured to serve as positive and negative controls, respectively. Released fluorescently labeled calcium phosphate in the media supernatant was detected and quantified in a multimode microplate reader FlexStation 3 (Molecular Devices).

Osteoclast differentiation was assessed by quantification of multinucleated cells following nuclear staining of RAW264.7 cells with NucBlue Live Cell Stain (Invitrogen) after 5 days of co-culture. Images at 40 × magnification of fluorescent (350 nm) were acquired and cells with distinct three or more nuclei were manually quantified (*n* = 8 wells/condition) using an inverted microscope EVOS cell imaging system.

### Statistics

All experiments were performed at least in triplicate. For μCT analysis, mean volume measurements from each of the exposed tooth were normalized to the contralateral control tooth and data were presented as % void volume. For IHC, mean immunoreactive cell numbers from each sample were normalized to their contralateral control (*n* = 9–12/time point). For the osteoblast and osteoclast function assays, the data were normalized to results from control group (without co-culture).

Statistical significance in differences of gene expression between control and ablated mice was tested using student’s *t*-test. One-way analysis of variance (ANOVA) with Bonferroni’s post hoc test was used to evaluate the behavior and in vitro mineralization and resorption assay data. Two-way (ANOVA) test with post hoc Sidak’s test was used to evaluate statistical significance of the osteolytic lesion area. Unpaired t-tests were used to analyze the RNAscope and immunohistochemistry quantification of macrophages, lymphocytes and RANKL/Osteoprotegerin (OPG) ratio. Statistical significance was set at *p* < 0.05. All statistical analyses were performed using GraphPad Prism 7.0 (GraphPad).

## Results

### Validation of Nav1.8^+^ nociceptor ablation

**Fig. 1:**
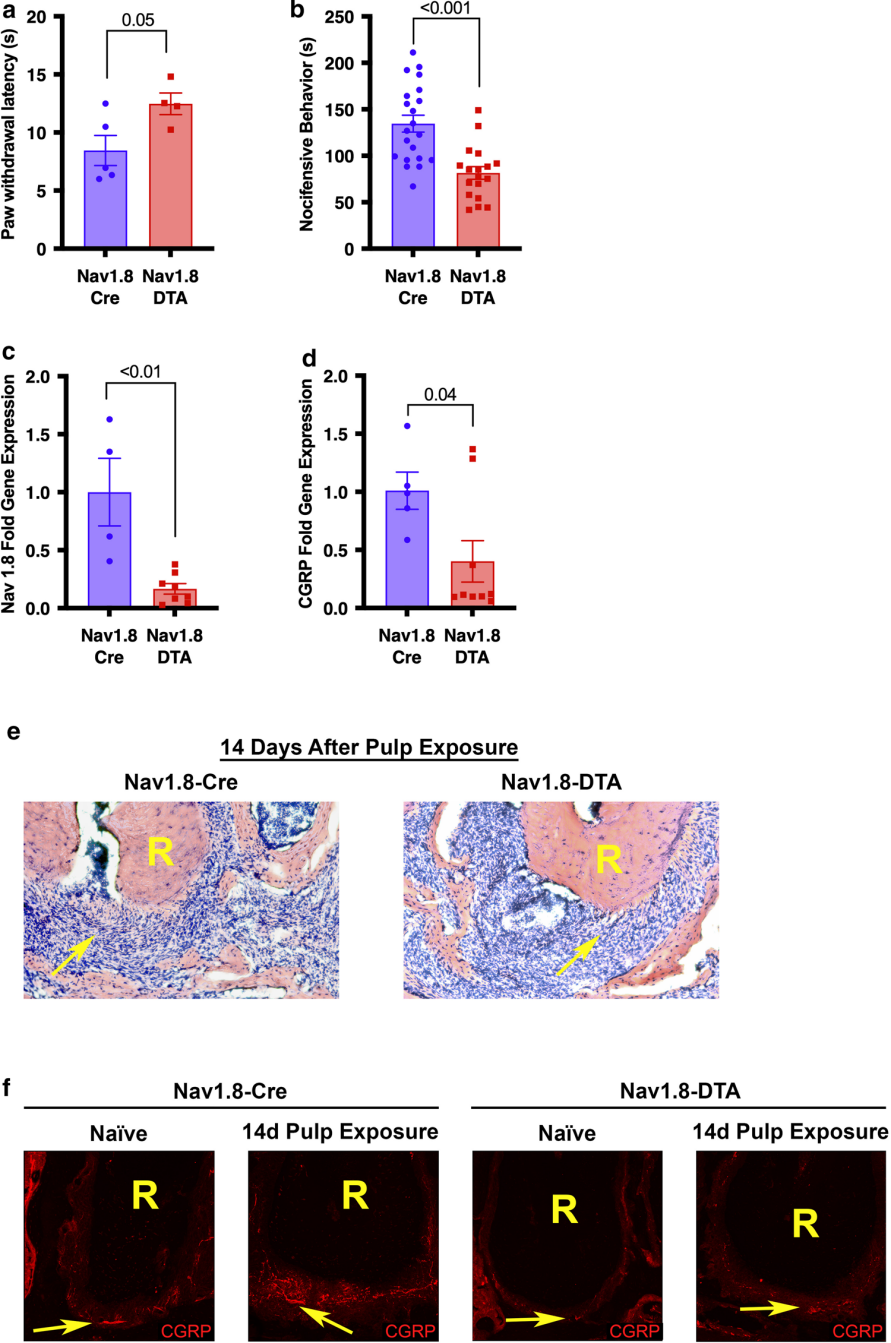
Behavioral, genetic, and histological profiles of Nav1.8-DTA and Nav1.8-Cre mice. Paw withdrawal latencies of radiant heat test (Hargreaves’s test) for Nav1.8-Cre and Nav1.8-DTA mice (**a**) (*n* = 4–5 per group). Time spent engaging in nocifensive behavior during capsaicin eye wipe test (**b**) (*n* = 18–21 per group). Nav1.8 gene expression in the TG normalized to cre-control (**c**) (*n* = 4–8 per group). CGRP gene expression in the TG normalized to cre-control (**d**) (*n* = 5–9 per group). Hematoxylin and eosin staining surrounding the root (R) of the tooth 14 days following pulp exposure in Nav1.8-Cre (left) and Nav1.8-DTA (right) mice (**e**). Staining CGRP^+^ nociceptors surrounding the root (R) of the tooth in Nav1.8-Cre (left) and Nav1.8-DTA (right) naïve and infected mice (**f**). Statistical analysis was performed using two-tailed unpaired *t* test. All graphs are shown as mean ± SEM

Thermal nociception tests (Hargreaves’s test) showed a significant increase in paw withdrawal latency in nociceptor-ablated mice (Nav1.8-DTA) compared to cre-control (Nav1.8-Cre) (*p* = 0.05) (Fig. 1a) (*n* = 4–5 per group). Nav1.8-DTA mice also spent significantly less time in nocifensive behavior following the capsaicin eye-wipe test (*p* < 0.001) (Fig. 1b) (*n* = 18–21 per group). There was marked reduction in the expression of Nav1.8 (*p* < 0.01) (Fig. 1c) (*n* = 4–8 per group) and CGRP (*p* = 0.04) (Fig. 1d) (*n* = 5–9 per group) mRNA in TG from Nav1.8-DTA compared to the Nav1.8-Cre mice.

Dental infection resulted in a robust cellular infiltrate at the inflammatory site in control mice (Fig. 1e; left) which increased with marked greater osteolytic area in Nav1.8-DTA mice (Fig. 1e; right). Next, the innervation status of the AP lesions and the contralateral healthy PDL space was evaluated by examining the expression of CGRP-immunoreactive (CGRP-IR, peptidergic nociceptor marker) fibers. Healthy teeth have rich innervation of nociceptors in Nav1.8-Cre mice that is substantially increased 14 days following infection due to neuronal sprouting (Fig. 1f; left). However, there was marked reduction in the density of CGRP-IR fibers in bone surrounding healthy teeth and within the AP lesion at 14 days following infection in the Nav1.8-DTA mice (Fig. 1f; right).

### Quantification of osteolytic lesions and key cytokines in apical periodontitis

**Fig. 2:**
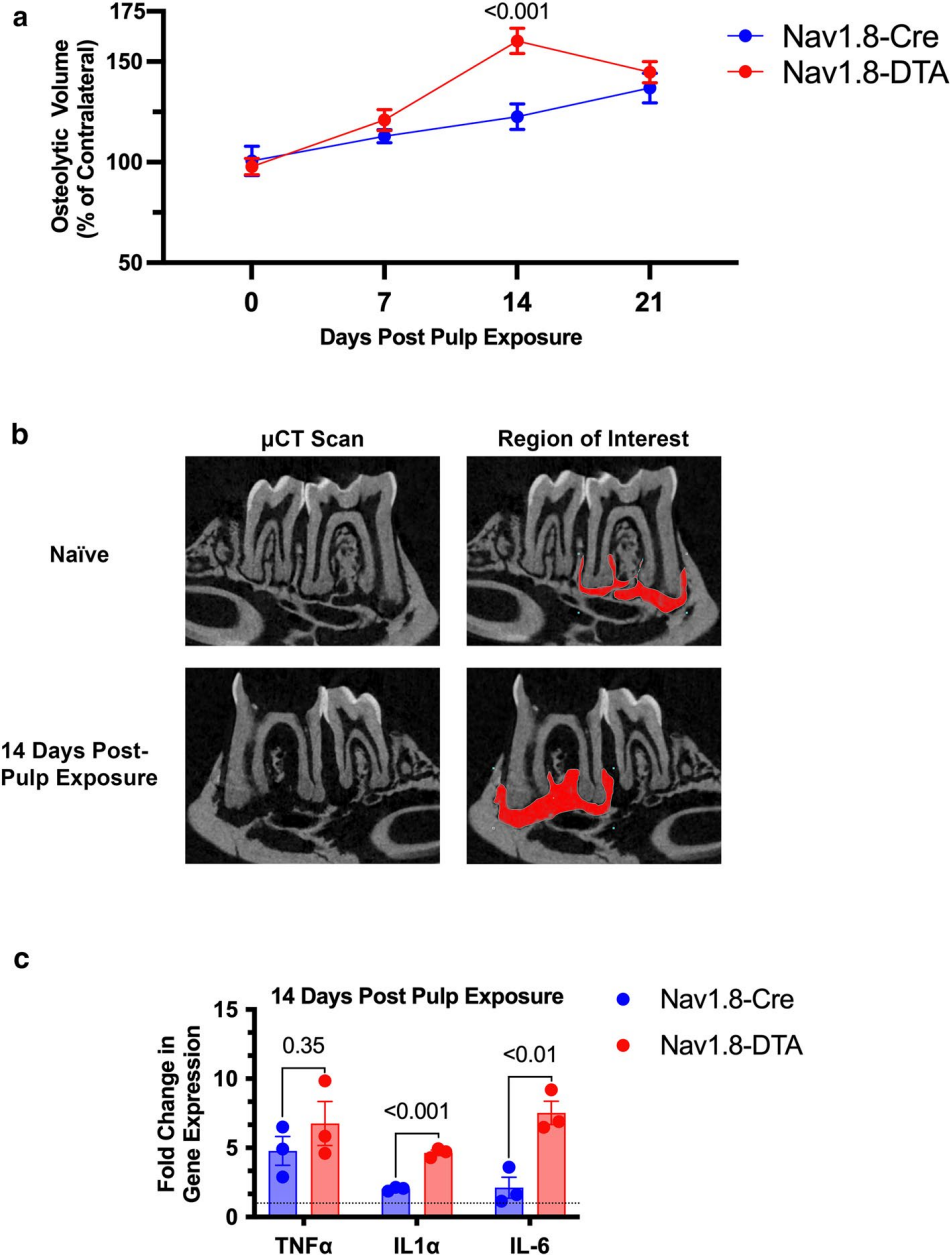
Nociceptor-ablation enhances AP osteolytic lesion development and expression of inflammatory mediators*.* Lesion development and progression in Nav1.8-Cre and Nav1.8-DTA mice measured by volume of apical voids via μCT normalized to contralateral side (**a**) (*n* = 6–7 per group). Representative images of μCT scans in naïve (upper left) and infected (lower left) molars. Representative slice of manual contouring of the void space around the apical third (0.3 mm from apex) for naïve (upper right) and infected (lower right) molars (**b**). Expression of TNF⍺, IL-1⍺, and IL-6 in the periapical lesion for Nav1.8-Cre and Nav1.8-DTA mice 14 days following pulp exposure normalized to contralateral side **(c)** (*n* = 3 per group). Statistical analysis was performed using a two-way ANOVA for (**a**) and multiple unpaired *t* test for (**c**). All graphs are shown as mean ± SEM

All mice (*n* = 6–7/time point) exhibited evident apical lesions detected by μCT starting on day 7 (Fig. 2a). However, Nav1.8-DTA animals had significantly larger lesions at day 14 than Nav1.8-Cre animals (Fig. 2a, b) (*p* < 0.001).

After 14 days post pulp exposure (infection), there were no differences in tumor necrosis factor alpha (TNF⍺) gene expression in the periapical lesion between Nav1.8-Cre and Nav1.8-DTA mice with both showing approximately a five-fold increase compared to healthy tissues (*p* = 0.35) (Fig. 2c) (*n* = 3 per group). Also, the expression of interleukin 1 alpha (IL-1α) and interleukin 6 (IL-6) were increased approximately two-fold compared to healthy tissues in Nav1.8-Cre mice (Fig. 2c). In Nav1.8-DTA mice, there was a significant increase in the expression of both IL-1α (*p* < 0.001) and IL-6 (*p* < 0.01) to approximately five- and seven-fold, respectively (Fig. 2c).

### Immunohistochemical analysis T lymphocytes, macrophages, and RANKL/OPG in periapical lesion

**Fig. 3:**
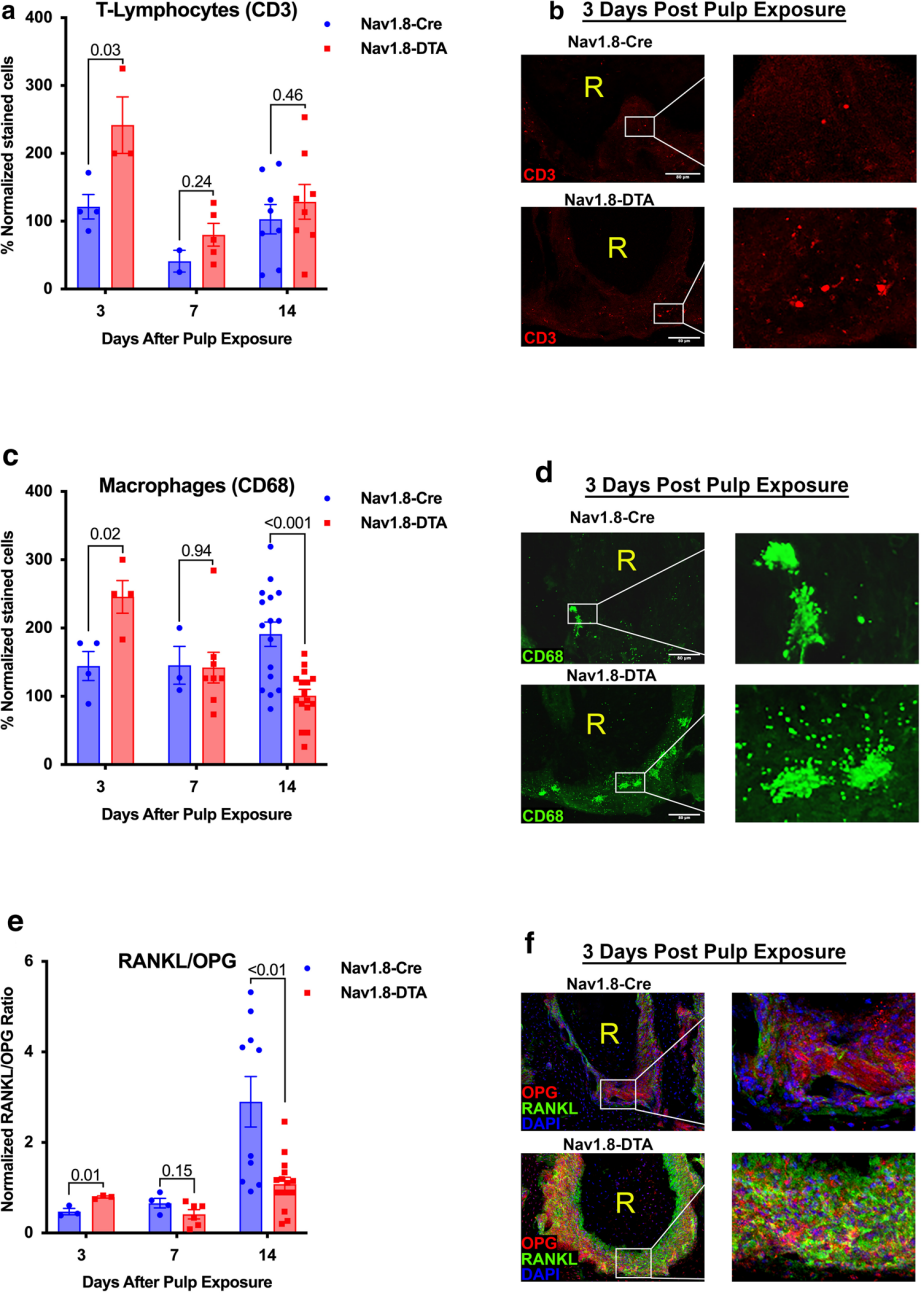
Nociceptor-ablation increases early infiltration of T lymphocytes and macrophages and expression of RANKL/OPG in AP periapical lesion*.* Quantification of CD3^+^ T Lymphocytes at days 3, 7, and 14 (**a**) (*n* = 2–8 per group). Representative 20 × confocal images of day 3 expression in Nav1.8-Cre (upper) and Nav1.8-DTA (lower) mice of CD3^+^ T Lymphocytes (**b**). Quantification of CD68^+^ macrophages at days 3, 7, and 14 (**c**) (*n* = 3–16 per group). Representative 20 × confocal images of day 3 expression in Nav1.8-Cre (upper) and Nav1.8-DTA (lower) mice of CD68^+^ macrophages (**d**). Quantification of RANKL/OPG expression at days 3, 7, and 14 (**e**) (*n* = 3–15 per group). Representative 20 × confocal images of day 3 expression in Nav1.8-Cre (upper) and Nav1.8-DTA (lower) mice of RANKL/OPG expression (**f**). Statistical analysis was performed using two-tailed unpaired *t* tests per target, per time point. All graphs are shown as mean ± SEM

Quantification of CD3^+^ T-lymphocytes and CD68^+^ macrophages in the apical lesion was performed with IHC with characterized antibodies. CD3^+^ cells were significantly more present in lesions of Nav1.8-DTA animals at 3 days (*p* = 0.03) compared to control with no difference observed at 7 or 14 days (*p* = 0.24, *p* = 0.46, respectively) (Fig. 3a, b) (*n* = 2–8 per group). Similarly, Nav1.8-DTA mice showed early higher infiltration of CD68^+^ cells at 3 days (*p* = 0.02), but that difference was not present at 7 days (*p* = 0.94) (Fig. 3c, d) (*n* = 3–16 per group). However, Nav1.8-Cre mice exhibited greater expression of CD68^+^ cells after 14 days (*p* < 0.001).

The ratio of cells expressing RANKL and OPG has been widely used to denote the status of dynamic bone metabolism [35]. Commonly, a high ratio of RANKL/OPG represents active osteolytic activity. In IHC studies, there was a significant higher RANKL/OPG ratio within apical lesions in Nav1.8-DTA animals at 3 days post pulp exposure (~ two-fold) (*p* = 0.01) (Fig. 3e, f). While no significant difference in the RANKL/OPG ratio was found between the two groups at 7 days (*p* = 0.15) (Fig. 3e), Nav1.8-DTA mice showed downregulation in the RANKL/OPG ratio compared to Nav1.8-Cre mice (~ two-fold) at 14 days (*p* < 0.01) (Fig. 3e).

### RNAscope mRNA analysis of T lymphocytes, macrophages, osteoblasts, and osteoclasts in periapical lesion

**Fig. 4:**
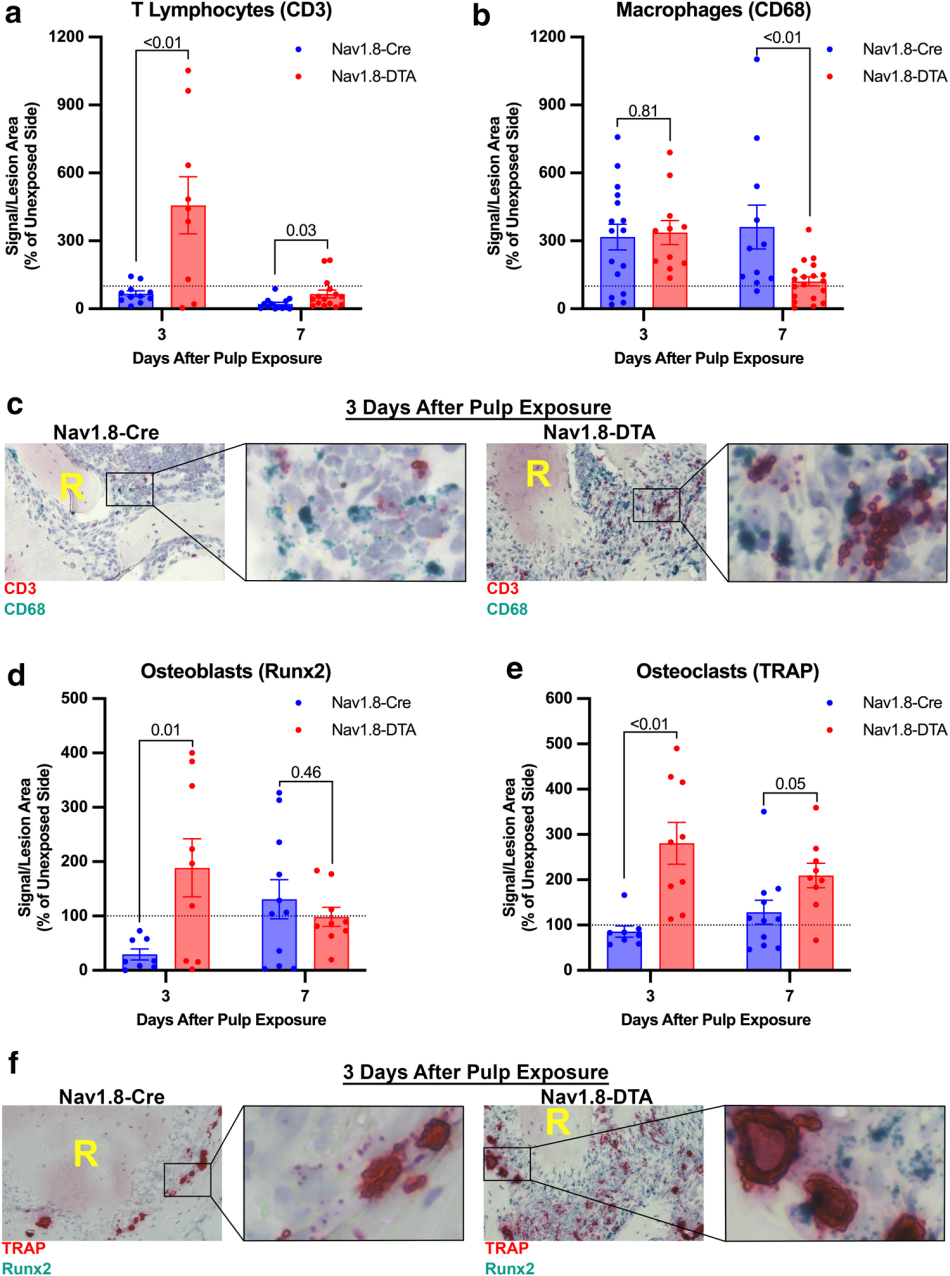
Nociceptor-ablation modulates mRNA expression of key immune and bone metabolism genes*.* Quantification of CD3^+^ T Lymphocytes at days 3 and 7 (**a**) (*n* = 8–16 per group). Quantification of CD68^+^ macrophages at days 3 and 7 (**b**) (*n* = 11–19 per group). Representative 40 × brightfield images of RNAscope staining for day 3 CD3^+^ T Lymphocytes (red) and CD68^+^ macrophages (green) for Nav1.8-Cre and Nav1.8-DTA mice (**c**). Quantification of Runx2^+^ osteoblasts at days 3 and 7 (**d**) (*n* = 8–11 per group). Quantification of TRAP^+^ osteoclasts at days 3 and 7 (**e**) (*n* = 8–11 per group). Representative 40 × brightfield images of RNAscope staining for day 3 TRAP^+^ osteoclasts (red) and Runx2^+^ osteoblasts (green) for Nav1.8-Cre and Nav1.8-DTA mice (**f**). Statistical analysis was performed using two-tailed unpaired *t* tests per target, per time point. Data are presented as area of signal per lesion area normalized to the contralateral side, and all graphs are shown as mean ± SEM

The mRNA expression of genes of interest was detected and quantified using RNAscope in situ hybridization. Within the periapical lesion, CD3 expression was significantly greater in Nav1.8-DTA mice at 3 and 7 days following pulp exposure (*p* < 0.01 and *p* = 0.03, respectively) (Fig. 4a, c) (*n* = 8–16 per group). Nevertheless, there was a significant lower expression of CD68 mRNA expression within AP in Nav1.8-DTA mice at day 7 (*p* < 0.01) with no significant difference (*p* = 0.81) in the early time point (3 days) compared to Nav1.8-Cre mice (Fig. 4b, c) (*n* = 11–19 per group).

Analysis of gene expression by RNAscope within the periapical lesion demonstrated that there is greater expression of Runx2 mRNA (expressed in osteoblasts) in Nav1.8-DTA mice 3 days following pulp exposure compared to Nav1.8-Cre (*p* = 0.01), whereas there is not a significant difference at 7 days (*p* = 0.46) (Fig. 4d, f) (*n* = 8–11 per group). Additionally, there was significantly greater gene expression of tartrate-resistant acid phosphatase (TRAP; expressed in osteoclasts) in Nav1.8-DTA lesions at both 3 and 7 days (*p* < 0.01, 0.05, respectively) (Fig. 4e, f) (*n* = 8–11 per group).

### Expression of osteocalcin and Ki67 within apical periodontitis

**Fig. 5:**
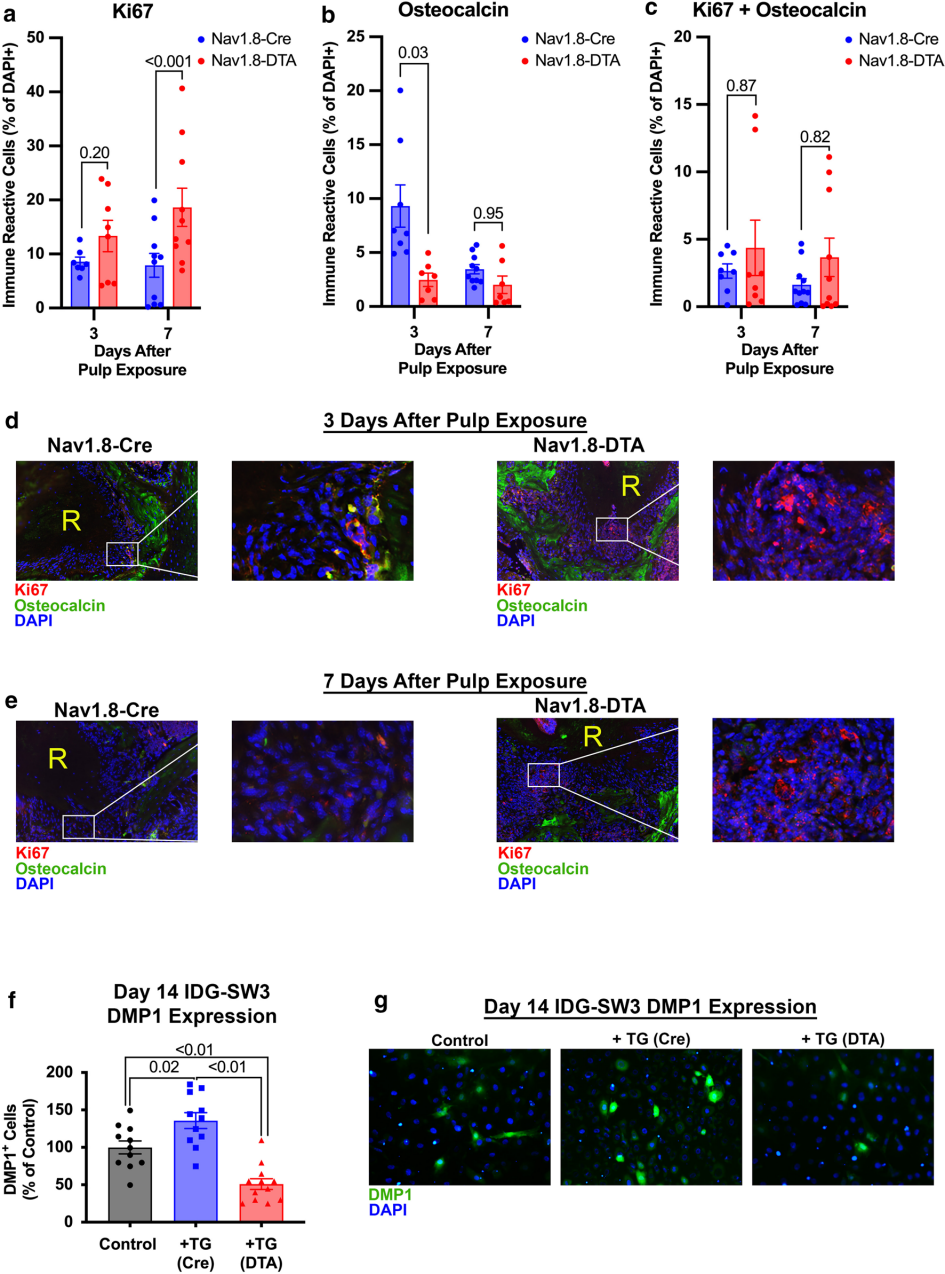
Nociceptor-ablation inhibits osteoblast differentiation*.* Quantification of Ki67^+^ progenitors at days 3 and 7 (**a**) (*n* = 7–10 per group). Quantification of Osteocalcin^+^ osteoblasts at days 3 and 7 (**b**) (*n* = 7–10 per group). Quantification of colocalized Ki67^+^ progenitors and Osteocalcin^+^ osteoblasts at days 3 and 7 (**c**) (*n* = 8–11 per group). Representative 20 × fluorescent images of Ki67 (red), osteocalcin (green), and DAPI (blue) staining surrounding the tooth root (R) for day 3 **(d)** and day 7 **(e)** samples. Quantification of DMP1^+^ IDG-SW3 cells after co-culture with control or Nav1.8-DTA TG neurons **(f)** (*n* = 11–12 per group) and representative 20 × images showing DMP1 expression (green) and DAPI (blue) **(g)**. Statistical analysis was performed using a two-way ANOVA for (**a–c**) and one-way ANOVA for (**f**). All graphs are shown as mean ± SEM

Quantification of the marker of proliferation Ki67 (Ki67)^+^ progenitor cells and osteocalcin^+^ osteoblasts was evaluated at 3 and 7 days following pulp exposure. Nociceptor ablation resulted in a greater expression of Ki67 within the lesion at 7 days (*p* < 0.001) but not at 3 days compared to control (*p* = 0.20) (Fig. 5a, d, e) (*n* = 7–10 per group). There was also a reduced expression of osteocalcin at 3 days in Nav1.8-DTA compared to Nav1.8-Cre mice (*p* = 0.03), but no significant differences at day 7 (*p* = 0.95) (Fig. 5b, d, e) (*n* = 7–10 per group). Finally, there was no difference in cells co-expressing both Ki67 and osteocalcin at day 3 or 7 (*p* = 0.87 and *p* = 0.82, respectively) (Fig. 5c, d, e) (*n* = 8–11 per group).

### Effect of TG neurons on osteoblast function

Co-culture of IDG-SW3 osteoblast precursor cells with TG neurons from Nav1.8-DTA mice resulted in decreased expression of DMP1 compared to cultures with Nav1.8-Cre TG neurons (*p* < 0.01) and with no neurons (*p* < 0.01) (Fig. 5f, g). On the contrast, IDG-SW3 cells co-cultured with control TG neurons expressed greater levels of DMP1 compared to control IDG-SW3 cells (*p* = 0.02) (Fig. 5f, g) (*n* = 11–12 per group).

**Fig. 6:**
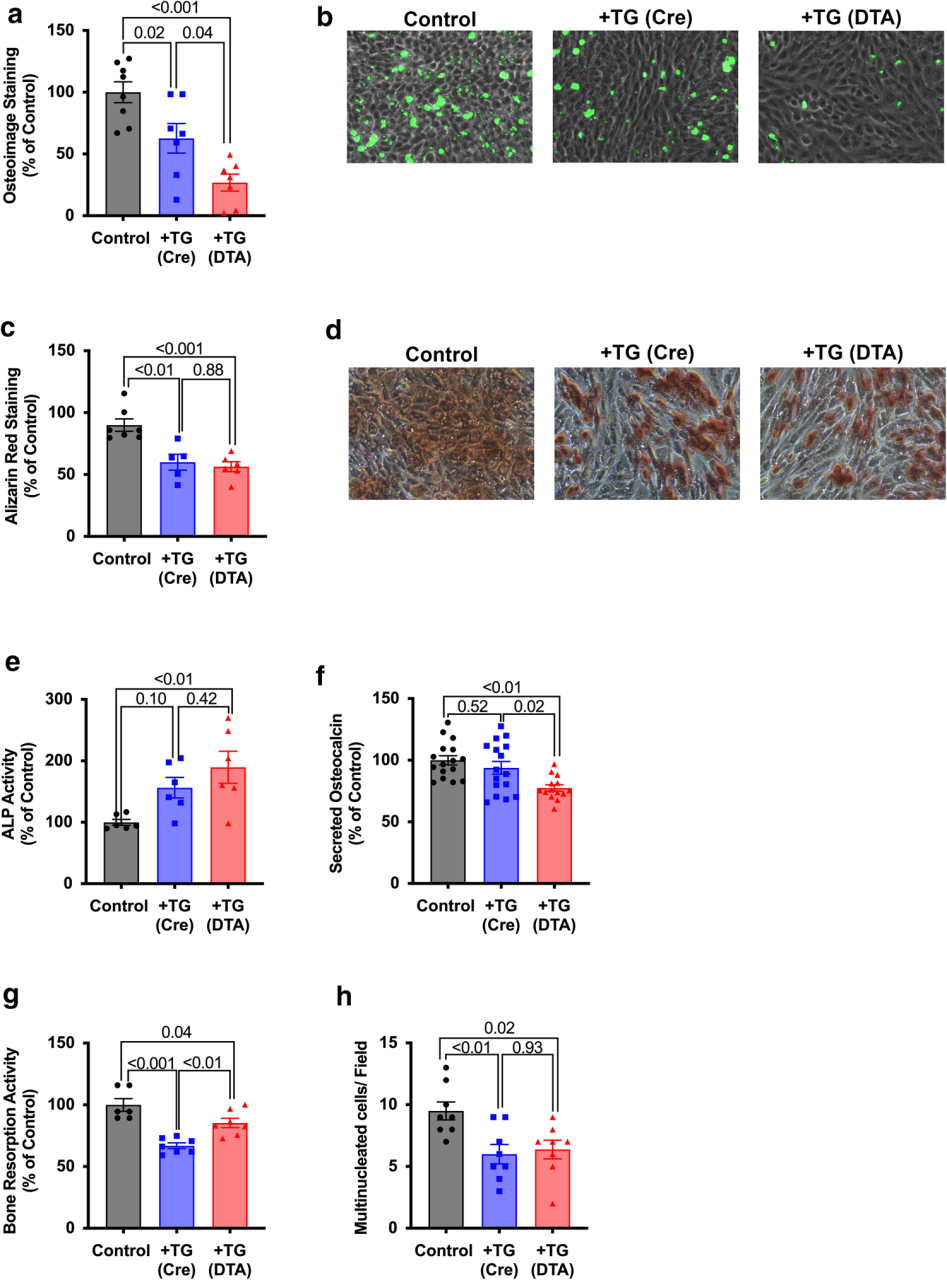
Nociceptors enhance osteoblast functioning and inhibit osteoclast activity*.* Quantification of Osteoimage fluorescent hydroxyapatite staining (**a**) (*n* = 7–8 per group). Representative 20 × images frsom MC3T3-E1 cultured in osteogenic differentiation media with trigeminal ganglia from Nav1.8-Cre [+ TG (cre)] or Nav1.8-DTA [+ TG (DTA)] mice for Osteoimage (**b**). Quantification of Alizarin Red calcium staining (**c**) (*n* = 5–7 per group). Representative 20 × images from MC3T3-E1 cultured in osteogenic differentiation media with trigeminal ganglia from Nav1.8-Cre (+ TG (cre)) or Nav1.8-DTA (+ TG (DTA)) mice for Alizarin Red (**d**). Data are presented as either relative fluorescence or absorbance as percent of control (i.e., MC3T3-E1 cells cultured alone). Quantification of alkaline phosphatase activity and secreted osteocalcin of MC3T3-E1 cells after co-culture with Nav1.8-Cre or Nav1.8-DTA TG neurons (**e** and **f**, respectively) (*n* = 6 per group and *n* = 14–16 per group, respectively). Data are presented as fluorescent (ALP activity) and absorbance (osteocalcin expression) readings normalized to control. Quantification of RAW264.7 bone resorption activity, presented as fluorescent reading normalized to control (**g**) (*n* = 6–7 per group). Quantification of multinucleated osteoclasts, represented as total number of multinucleated cells per field of view (**h**) (*n* = 8 per group). Statistical analysis was performed using a two-way ANOVA. All graphs are shown as mean ± SEM

Calvaria osteoblast precursor cells co-cultured with primary TG neuronal cultures resulted in the significant inhibition of their mineralization potential, as evaluated with Osteoimage (63 ± 12% of control; *p* = 0.02) (Fig. 6a, b) and Alizarin Red (*p* < 0.01) (Fig. 6c, d). This inhibition is further and significantly greater when osteoblasts were co-cultured with primary trigeminal cultures from Nav1.8-DTA mice (27 ± 6.8% of control; *p* = 0.04) (Fig. 6a, b), whereas there were no significant differences between Nav1.8-cre and Nav1.8-DTA TG culture for Alizarin Red staining (*p* = 0.88) (Fig. 6c, d). Additionally, co-culture with trigeminal ganglia from control animals had no effect on alkaline phosphatase (ALP) activity (*p* = 0.10), whereas cells co-cultured with Nav1.8-DTA trigeminal ganglia had significant increase in ALP activity (*p* < 0.01) (Fig. 6e) (*n* = 6 per group). Finally, Nav1.8-DTA TG cultures reduced the amount of osteocalcin secreted in the media compared to both control TG co-cultures and cells cultured alone (*p* = 0.02 and *p* =  < 0.01, respectively) (Fig. 6f) (*n* = 14–16 per group).

### Effect of TG neurons on in vitro bone resorption activity

Co-culture of murine osteoclast precursor cells (RAW264.7) with primary TG neurons inhibited resorption activities compared to control (67 ± 6.1% of control; *p* < 0.001) This inhibition was partially reversed by co-culture with neurons from Nav1.8-DTA mice (85 ± 10% of control; *p* < 0.01) but was still lower compared to Nav1.8-Cre (*p* = 0.04) (Fig. 6g) (*n* = 6–7 per group).

Upon counting the number of multinucleated osteoclast-like cells, co-cultures with control and Nav1.8-DTA TG neurons had a reduced number of multinucleated cells compared to Nav1.8-Cre (*p* = 0.01, 0.02, respectively) (Fig. 6h). Interestingly, there were no significant differences in the number of osteoclast-like cells between induced RAW264.7 cells co-cultured with TG neurons from control and Nav1.8-DTA (nociceptor ablated) TG cultures (*p* = 0.93) (Fig. 6h) (*n* = 8 per group).

### Transcriptomic analysis of osteoblasts co-cultured with TG neurons

**Fig. 7:**
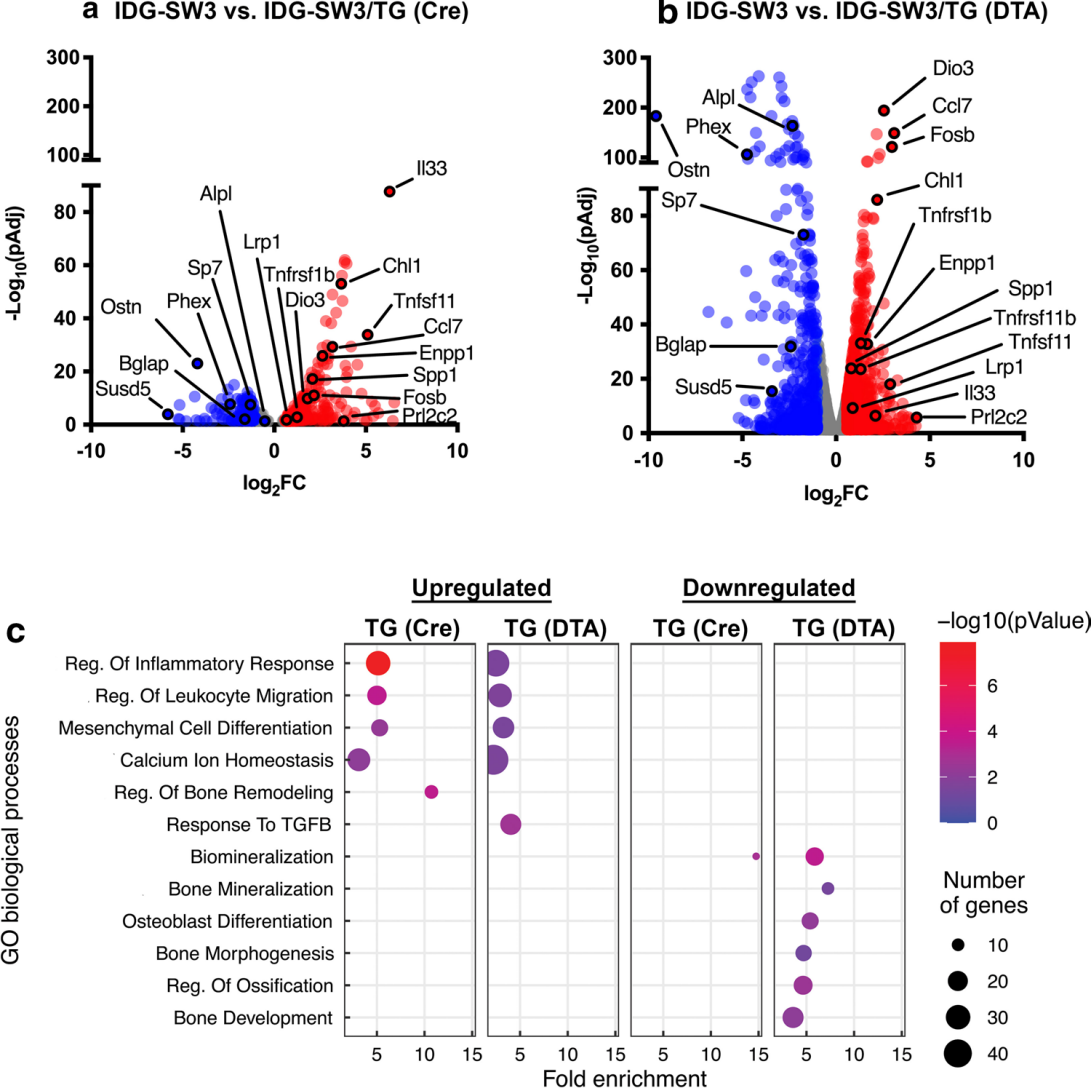
Nociceptors enhance expression of osteoblast differentiation genes*.* Volcano plots of significant (pAdj < 0.05) differentially expressed genes (DEGs) between control IDG-SW3 cells and either Nav1.8-Cre TG co-cultures (**a**) or Nav1.8-DTA TG co-cultures (**c**) plotted as log_2_ Fold Change (FC) against -log(pAdj) (*n* = 2 per group). DEGs with FC > 1.5 are in red, and those where FC < 0.5 are plotted in blue, and all other DEGs are colored gray. Gene ontology analyses of upregulated and downregulated (**c**) genes for Nav1.8-Cre and Nav1.8-DTA are plotted as Fold Enrichment for each biological process, where color correlates to -log(*p*Value) and size is related to the number of genes enriched in each process. Pairwise comparisons were performed using the Benjamin-Hodjberg test

Co-culture of IDG-SW3 osteoblast precursor cells with TG neurons from control mice for 14 days resulted in 494 genes that were differentially expressed (being 369 upregulated and 125 downregulated) compared to IDG-SW3 cells cultured alone (FC > 1.5, padj < 0.05) (Fig. 7a). When osteoblasts were co-cultured with Nav1.8-DTA TG, there were 1680 differentially expressed genes (DEGs), with 974 upregulated and 706 downregulated (FC > 1.5, padj < 0.05) (Fig. 7b). There were 203 genes that were commonly upregulated, and 92 genes that were commonly downregulated in both co-cultures with TG neurons from control and Nav1.8-DTA mice (Supplemental Fig. 1a, b, respectively). It is noteworthy that several markers of osteoblast differentiation were significantly more downregulated in co-cultures with Nav1.8-DTA neurons. This included a 27-fold downregulation in the expression of phosphate regulating endopeptidase homolog X-linked (PHEX), a marker of later osteoblastic differentiation, when co-cultured with Nav1.8-DTA neurons compared to a 5.2 reduction seem in control TG co-cultures. Importantly, there was a 5.4-fold downregulation of osteocalcin gene [bone gamma-carboxyglutamate protein (BGLAP)] in co-cultures with Nav1.8-DTA neurons. Next, alkaline phosphatase (ALPL) gene expression decreased by five-fold in osteoblast precursors cultured with Nav1.8-DTA neurons. Osteocrin (Ostn), an osteoblast-specific marker [36, 37], had the significant downregulation of 783-fold in cells co-cultured with nociceptor ablated neurons compared to cells cultured with control neurons. In addition, other putative osteoblast differentiation markers, including Sp7 Transcription Factor (SP7) and Secreted Phosphoprotein-1 (SPP1), had lower expression in cells co-cultured with Nav1.8-DTA TG neurons (Fig. 7b).

Gene ontology analyses revealed nociceptors regulate enrichment of select biological processes within IDG-SW3 TG neuronal co-cultures (Fig. 7c). Within genes that were upregulated, osteoblast precursors with Nav1.8-DTA TG neurons exhibited a greater number of genes compared to control for processes including “Regulation of Inflammatory Response” (36 vs. 30 genes) and “Regulation of Leukocyte Migration” (28 vs. 19 genes). Interestingly, only Nav1.8-Cre, not Nav1.8-DTA, cultures exhibited the upregulation of genes in “Regulation of Bone Remodeling” (11 genes). Only Nav1.8-DTA cultures had an enrichment of “Response to Transforming growth factor beta (TGF-β)” (22 genes). Within downregulated genes, co-culture with Nav1.8-DTA TG neurons exhibited greater numbers of genes in biological processes including “Biomineralization” (17 vs. 8 genes) compared to Nav1.8-Cre cultures. Importantly, only Nav1.8-DTA cultures demonstrated downregulation of genes in “Bone Mineralization” (10 genes), “Osteoblast Differentiation” (15 genes), “Bone Morphogenesis” (14 genes), “Regulation of Ossification” (18 genes), and “Bone Development” (22 genes).

## Discussion

Apical periodontitis is a prevalent inflammatory disease resulting from dental infection that affects millions worldwide [38]. Pain, loss of function, inflammation and localized bone loss are hallmark features of this cranio-skeletal disease. Although human teeth are one of the tissues most densely with nociceptors [39], the function of the specialized sensory is believed to be mainly sensorial and their role in regulating the cellular events in apical periodontitis is largely unknown. In this study, using a transgenic approach to achieve the conditional ablation of nociceptors (marked as Nav1.8^+^ neurons), we found that nociceptors regulate early stages of the apical periodontitis initiation and progression by suppressing an initial immune response, which are seen as reduced influx of macrophages and lymphocytes and lower expression of IL-1⍺ and IL-6, promoting osteoblast differentiation and inhibiting osteoclast differentiation and its mediated resorption.

We had previously demonstrated that pharmacological ablation of a subpopulation of nociceptors (TRPV1^+^ neurons) resulted in accelerated bone loss in a model of apical periodontitis in rats [8]. It is well established that bone homeostasis is result of the net function between osteoblasts and osteoclasts, and that this balance can be shifted towards greater resorption due to inflammation. In this study, a transgenic approach allowed for the targeted ablation of nociceptors to evaluate their participation in the cellular events that mediate bone loss in apical periodontitis. This approach prevents possible off-target effects of large doses of capsaicin in neonatal rats [18]. In addition, this transgenic approach is well-characterized and successfully used in investigate the participation of sensory innervation in different biological processes [18, 40, 41]. Nav1.8-DTA mice and the control mice showed normal development with no aberrant systemic or dental manifestations including similar bone density and morphology. The successful ablation of a large subset of nociceptors was confirmed by behavior assays with mice displaying longer paw withdrawal latencies to radiant heat stimulus, and significantly less time spent in nocifensive behavior upon application of a capsaicin solution in the eye. At the molecular level, there was a dramatic loss of Nav1.8 and CGRP mRNA in the trigeminal ganglia, representing a significant loss of nociceptors since Nav1.8 is preferentially expressed in nociceptors [17] and CGRP is expressed primarily in a subpopulation of nociceptors (peptidergic) [15]. At the cellular level, the loss of innervation could be confirmed by immunohistochemistry with dramatic reduction of CGRP staining in nerve fibers innervating the periodontal ligament between teeth and the surrounding bone. This innervation pattern is known to have considerable plasticity with robust sprouting of nociceptors within the site of apical periodontitis following infections [22]. This sprouting was indeed observed in control mice but largely abolished in Nav1.8-DTA mice seen as loss of CGRP-positive nerve fibers. Collectively, the results demonstrated that the used transgenic approach resulted in great reduction of nociceptors that could be observed within the tissue at the site of apical periodontitis.

Models of infection-induced apical periodontitis have been widely used to study the initiation, progression and maintenance of apical periodontitis in mice [42, 43]. The kinetics of bone loss has been previously reported with the use of either histology or micro-CT as dependent measure [44]. Similar to previous studies, we found a steady increase in osteolytic activity in control mice with a plateau of maximum bone loss after 21 days following pulp exposures to the oral environment (infection). Ablation of nociceptors resulted in larger osteolytic lesions osteolytic lesions developed at earlier time points as detected by histochemical staining and micro-CT.

The development of AP lesions is marked by an increase in inflammatory mediators, including IL-1⍺ expression that is found increased in both animal models [45] and human tissues [46], and that bone resorption is significantly blocked by infusion with neutralizing antibodies against IL-1⍺, confirming its pivotal role in mediating bone resorption in this disease [45]. In this study, we also detected a significant increase of IL-1⍺ expression that was dramatically increased in Nav1.8-DTA mice. Similarly, IL-6 is a pro-inflammatory mediator that has been implicated in apical periodontitis with levels directly proportional the sizes of the osteolytic lesions in both animal models and human tissues [47]. We found a robust upregulation in IL-6 mRNA expression that was dramatically increased in nociceptor ablated mice. This increase in the inflammatory status in apical periodontitis seen in nociceptor ablated mice was also observed by an early increase macrophages and lymphocytes detected by both immunohistochemistry and RNAscope in situ hybridization. Macrophages are important specialized innate immune cells which constitutes the major cell population in AP lesion and are known to synthesize and release pro-resorptive cytokines [6]. They are known to mediate key events during the early defense mechanism and during transitioning to adaptive response by activating T-lymphocytes [5]. In a similar manner, the anticipation of T-lymphocytes increase seen with nociceptor ablation may underlie an important mechanism since they are known secrete RANKL early during AP development [48]. Interestingly, a study using the transgenic ablation of nociceptors to investigate their function in regulating immunological responses in lung infections also found increased inflammatory markers and greater immune cell infiltrate following nociceptor ablation [49]. Thus, nociceptors inhibit macrophage and lymphocyte infiltration in the early stages of AP development and downregulate the expression of IL-1⍺ and IL-6.

Osteoclastogenesis and bone resorption is initiated and maintained by the agonist action of RANKL on its receptor in osteoclast precursor cells. Several inflammatory mediators, also known to be present in AP, have been shown to be co-activators of this process such as TNF-α and IL-1 [50]. This effect is counteracted by OPG that acts as a decoy soluble receptor sequestering available RANKL. Thus, the overall resorptive activity is highly dependent on RANKL/OPG ratio [51]. Indeed, we found concomitant upregulation of RANKL/OPG expression with the phase of active osteolytic activities of the lesion. This is in agreement with previous study evaluating the levels of RANKL/OPG ratio in relation to the bone destruction and the expression of pro-resorption inflammatory mediators [35]. They also found that in naïve animals, the RANKL/OPG ratio peaked at 14–21 days post-AP induction and then decreased afterward during chronic stage. However, this increase in RANKL/OPG ratio was significantly anticipated in Nav1.8-DTA mice with peak seen at 7 days agreeing with the quantification of bone loss in ablated mice and correlated with the increased early inflammatory infiltrate and the increase in TRAP mRNA detected by in situ hybridization. Further, the direct modulation of neurons on osteoclasts was investigated by co-culture experiments of RAW264.7 cells with trigeminal neurons using an established bone resorption assay [34]. In this assay, RANKL and lipolysaccharides (LPS) were used to stimulate the osteoclastic differentiation while in presence of trigeminal cultures from control or Nav1.8-DTA mice. It is noteworthy, that besides promoting RAW264.7 differentiation in vitro [52], LPS is one of the primary bacterial antigens in dental infections with direct correlation with symptoms and size of bone resorptive lesions in AP [53]. Our results revealed that TG neurons inhibit osteoclastic activities were inhibited by TG neurons from control mice, but this effect was partially reversed in co-cultures with TG from Nav1.8-DTA mice. This effect does not appear to be due to reduced differentiation since there was not a difference in number of multinucleated cells between both co-cultures. Instead, the results represent a reduction in osteolytic activity mediated by soluble factors released from neuronal cultures in the semipermeable culture inserts. Although the neuronal derived factors mediating this effect were not identified, there is evidence that CGRP suppresses the differentiation and function of osteoclasts [54]. Collectively, we demonstrated that nociceptors downregulate osteoclastic function.

There is increasing evidence that support an intimate cross-talk between sensory innervation and osteoblasts in both bone homeostasis [55] and in pathological processes that result in the dysregulation of bone turnover [56]. In this study, an early increase in the osteoblastic precursor RUNX2 mRNA was detected at 3 days post infection in Nav1.8-DTA mice. Interestingly, this increase was mirrored by a decrease in osteocalcin-positive cells and the progressive increase of Ki67 + positive cells without the increase in co-expression between these two markers. These results suggest that nociceptors promote early differentiation of osteoblasts and the commitment of rapidly dividing progenitor cells (Ki67 +) [57] into an osteoblast phenotype (osteocalcin +) in apical periodontitis. Indeed there is evidence that sensory innervation can drive the commitment of undifferentiated mesenchymal cells The reporter osteoblast precursor cell line IDG-SW3 was used co-culture experiments to monitor the expression of DMP-1 by GFP, and due to its well-established genetic profile as it transition from early osteoblasts to late osteocytes [58]. Co-culture with TG neurons from control mice resulted in increased expression of DMP-1 that was dramatically decreased when cells were co-cultured with TG neurons from nociceptor ablated mice. RNA sequencing revelated that several genes related to osteoblast differentiation and function were significantly downregulated in cells co-cultured with nociceptor ablated TG neurons. Notably, the downregulation of the putative osteoblast marker BGLAP (osteocalcin) and the mature osteoblast/osteocyte marker Phex [59] supports that differentiation into mature osteoblasts was halted when co-cultured with Nav1.8-DTA TG neurons. Collectively, the transcriptomic analysis of IDG-SW3 co-culture experiments shown a robust downregulation in osteoblast differentiation and genes involved in mineralization and bone regulation by Nav1.8-DTA neurons compared to cells exposed to TG neurons from control mice. Evidence of the direct stimulation of osteoblast differentiation by nociceptors was also obtained by of murine calvaria osteoblast precursors, MC3T3-E1 [60] with TG neurons. Ablation of nociceptors resulted in reduced formation of hydroxyapatite deposits after 7 days in culture and reduced detection of secreted osteocalcin. Interestingly, there were no differences in calcium deposit staining by alizarin red and a greater alkaline phosphatase (ALP) activity was detected in co-cultures with Nav1.8-DTA neurons. This increase in ALP activity detected after 7 days in presence of differentiation factors is likely related to younger osteoblasts still entering the secretory phase, known to have higher ALPL expression [61], due to their retarded differentiation in absence of nociceptors. These effects are likely mediated by an entourage of secretory factors that have not yet been identified. Altogether, these experiments demonstrate that nociceptors promote osteoblast differentiation and mineralization in vivo and in vitro.

In conclusion, our findings demonstrate, for the first time, that TG nociceptors regulate the development of AP by immunomodulation observed as suppression of immune cell infiltration and decrease in cytokine production and increase of osteoblast differentiation while reducing osteoclastic activities. This newly uncovered neuroimmune mechanism of nociceptor regulation of bone turnover due to infections has broad implications to understanding infection mediated osteolysis, and could become a therapeutic target for the treatment of AP to reduce inflammation, while maximizing osseous healing after adequate treatment and minimize the persistence of osteolytic lesions in refractory cases.

### Supplementary Information

Below is the link to the electronic supplementary material.

Supplemental Figure 1. Nociceptors selectively regulate differential expression of genes in osteoblast precursors.Venn diagrams of distinct and common differentially expressed genes (FC>1.5, padj<0.05) for upregulated (1a) and downregulated (1b) genes in IDG-SW3 cells cultured with either Nav1.8-Cre or Nav1.8-DTA TG neurons. (PNG 119 KB)

Supplementary file2 (DOCX 14 KB)

Supplementary file3 (DOCX 14 KB)

Supplementary file4 (DOCX 15 KB)

Supplementary file5 (DOCX 14 KB)

Supplementary file6 (DOCX 14 KB)

## Data Availability

The datasets generated during and/or analyzed during the current study are available in the GEO repository, https://www.ncbi.nlm.nih.gov/geo/query/acc.cgi?acc=GSE198091.

## References

[1] Kassebaum NJ, Smith AGC, Bernabe E, Fleming TD, Reynolds AE, Vos T (2017). Global, regional, and national prevalence, incidence, and disability-adjusted life years for oral conditions for 195 countries, 1990–2015: a systematic analysis for the global burden of diseases, injuries, and risk factors. J Dent Res.

[2] Gomes BP, Lilley JD, Drucker DB (1996). Associations of endodontic symptoms and signs with particular combinations of specific bacteria. Int Endod J.

[3] Kakehashi S, Stanley HR, Fitzgerald RJ (1965). The effects of surgical exposures of dental pulps in germ-free and conventional laboratory rats. Oral Surg Oral Med Oral Pathol.

[4] Stashenko P (1990). Role of immune cytokines in the pathogenesis of periapical lesions. Endod Dent Traumatol.

[5] Kawashima N, Okiji T, Kosaka T, Suda H (1996). Kinetics of macrophages and lymphoid cells during the development of experimentally induced periapical lesions in rat molars: a quantitative immunohistochemical study. J Endod.

[6] Kawashima N, Stashenko P (1999). Expression of bone-resorptive and regulatory cytokines in murine periapical inflammation. Arch Oral Biol.

[7] Tomlinson RE, Christiansen BA, Giannone AA, Genetos DC (2020). The role of nerves in skeletal development, adaptation, and aging. Front Endocrinol (Lausanne).

[8] Austah ON, Ruparel NB, Henry MA, Fajardo RJ, Schmitz JE, Diogenes A (2016). Capsaicin-sensitive innervation modulates the development of apical periodontitis. J Endod.

[9] Takahashi N, Matsuda Y, Sato K, de Jong PR, Bertin S, Tabeta K (2016). Neuronal TRPV1 activation regulates alveolar bone resorption by suppressing osteoclastogenesis via CGRP. Sci Rep.

[10] Caterina MJ, Rosen TA, Tominaga M, Brake AJ, Julius D (1999). A capsaicin-receptor homologue with a high threshold for noxious heat. Nature.

[11] Gibbs JL, Melnyk JL, Basbaum AI (2011). Differential TRPV1 and TRPV2 channel expression in dental pulp. J Dent Res.

[12] Omari SA, Adams MJ, Geraghty DP (2017). TRPV1 channels in immune cells and hematological malignancies. Adv Pharmacol.

[13] Basbaum AI, Bautista DM, Scherrer G, Julius D (2009). Cellular and molecular mechanisms of pain. Cell.

[14] Lopes DM, Denk F, McMahon SB (2017). The molecular fingerprint of dorsal root and trigeminal ganglion neurons. Front Mol Neurosci.

[15] Patil MJ, Hovhannisyan AH, Akopian AN (2018). Characteristics of sensory neuronal groups in CGRP-cre-ER reporter mice: comparison to Nav1.8-cre, TRPV1-cre and TRPV1-GFP mouse lines. PLoS One.

[16] von Buchholtz LJ, Ghitani N, Lam RM, Licholai JA, Chesler AT, Ryba NJP (2021). Decoding cellular mechanisms for mechanosensory discrimination. Neuron.

[17] Akopian AN, Sivilotti L, Wood JN (1996). A tetrodotoxin-resistant voltage-gated sodium channel expressed by sensory neurons. Nature.

[18] Stirling LC, Forlani G, Baker MD, Wood JN, Matthews EA, Dickenson AH (2005). Nociceptor-specific gene deletion using heterozygous NaV1.8-Cre recombinase mice. Pain.

[19] Stashenko P, Teles R, D'Souza R (1998). Periapical inflammatory responses and their modulation. Crit Rev Oral Biol Med.

[20] Fukuda T, Takeda S, Xu R, Ochi H, Sunamura S, Sato T (2013). Sema3A regulates bone-mass accrual through sensory innervations. Nature.

[21] Jimenez-Andrade JM, Mantyh WG, Bloom AP, Xu H, Ferng AS, Dussor G (2010). A phenotypically restricted set of primary afferent nerve fibers innervate the bone versus skin: therapeutic opportunity for treating skeletal pain. Bone.

[22] Byers MR, Taylor PE, Khayat BG, Kimberly CL (1990). Effects of injury and inflammation on pulpal and periapical nerves. J Endod.

[23] Hargreaves K, Dubner R, Brown F, Flores C, Joris J (1988). A new and sensitive method for measuring thermal nociception in cutaneous hyperalgesia. Pain.

[24] Diogenes A, Patwardhan AM, Jeske NA, Ruparel NB, Goffin V, Akopian AN (2006). Prolactin modulates TRPV1 in female rat trigeminal sensory neurons. J Neurosci.

[25] Schmittgen TD, Livak KJ (2008). Analyzing real-time PCR data by the comparative C(T) method. Nat Protoc.

[26] Patwardhan AM, Berg KA, Akopain AN, Jeske NA, Gamper N, Clarke WP (2005). Bradykinin-induced functional competence and trafficking of the delta-opioid receptor in trigeminal nociceptors. J Neurosci.

[27] Austah O, Widbiller M, Tomson PL, Diogenes A (2019). Expression of neurotrophic factors in human dentin and their regulation of trigeminal neurite outgrowth. J Endod.

[28] Henriquez B, Bustos FJ, Aguilar R, Becerra A, Simon F, Montecino M (2013). Ezh1 and Ezh2 differentially regulate PSD-95 gene transcription in developing hippocampal neurons. Mol Cell Neurosci.

[29] Nicole O, Ali C, Docagne F, Plawinski L, MacKenzie ET, Vivien D (2001). Neuroprotection mediated by glial cell line-derived neurotrophic factor: involvement of a reduction of NMDA-induced calcium influx by the mitogen-activated protein kinase pathway. J Neurosci.

[30] Mecklenburg J, Zou Y, Wangzhou A, Garcia D, Lai Z, Tumanov AV (2020). Transcriptomic sex differences in sensory neuronal populations of mice. Sci Rep.

[31] Mi H, Muruganujan A, Huang X, Ebert D, Mills C, Guo X (2019). Protocol Update for large-scale genome and gene function analysis with the PANTHER classification system (v.14.0). Nat Protoc.

[32] Bonnot T, Gillard MB, Nagel DH (2019). A simple protocol for informative visualization of enriched gene ontology terms. Bio-Protocol..

[33] Matsuo S, Ichikawa H, Silos-Santiago I, Kiyomiya K, Kurebe M, Arends JJ (2002). Ruffini endings are absent from the periodontal ligament of trkB knockout mice. Somatosens Mot Res.

[34] Miyazaki T, Miyauchi S, Anada T, Imaizumi H, Suzuki O (2011). Evaluation of osteoclastic resorption activity using calcium phosphate coating combined with labeled polyanion. Anal Biochem.

[35] Kawashima N, Suzuki N, Yang G, Ohi C, Okuhara S, Nakano-Kawanishi H (2007). Kinetics of RANKL, RANK and OPG expressions in experimentally induced rat periapical lesions. Oral Surg Oral Med Oral Pathol Oral Radiol Endod.

[36] Thomas G, Moffatt P, Salois P, Gaumond MH, Gingras R, Godin E (2003). Osteocrin, a novel bone-specific secreted protein that modulates the osteoblast phenotype. J Biol Chem.

[37] Bord S, Ireland DC, Moffatt P, Thomas GP, Compston JE (2005). Characterization of osteocrin expression in human bone. J Histochem Cytochem.

[38] Tiburcio-Machado CS, Michelon C, Zanatta FB, Gomes MS, Marin JA, Bier CA (2021). The global prevalence of apical periodontitis: a systematic review and meta-analysis. Int Endod J.

[39] Hildebrand C, Fried K, Tuisku F, Johansson CS (1995). Teeth and tooth nerves. Prog Neurobiol.

[40] Udit S, Burton M, Rutkowski JM, Lee S, Bookout AL, Scherer PE (2017). Nav1.8 neurons are involved in limiting acute phase responses to dietary fat. Mol Metab.

[41] MacDonald DI, Luiz AP, Iseppon F, Millet Q, Emery EC, Wood JN (2021). Silent cold-sensing neurons contribute to cold allodynia in neuropathic pain. Brain.

[42] AlShwaimi E, Purcell P, Kawai T, Sasaki H, Oukka M, Campos-Neto A (2009). Regulatory T cells in mouse periapical lesions. J Endod.

[43] Balto K, White R, Mueller R, Stashenko P (2002). A mouse model of inflammatory root resorption induced by pulpal infection. Oral Surg Oral Med Oral Pathol Oral Radiol Endod.

[44] Balto K, Muller R, Carrington DC, Dobeck J, Stashenko P (2000). Quantification of periapical bone destruction in mice by micro-computed tomography. J Dent Res.

[45] Wang CY, Stashenko P (1993). The role of interleukin-1 alpha in the pathogenesis of periapical bone destruction in a rat model system. Oral Microbiol Immunol.

[46] Yang NY, Zhou Y, Zhao HY, Liu XY, Sun Z, Shang JJ (2018). Increased interleukin 1alpha and interleukin 1beta expression is involved in the progression of periapical lesions in primary teeth. BMC Oral Health.

[47] Azuma MM, Samuel RO, Gomes-Filho JE, Dezan-Junior E, Cintra LT (2014). The role of IL-6 on apical periodontitis: a systematic review. Int Endod J.

[48] Silva MJ, Kajiya M, AlShwaimi E, Sasaki H, Hong J, Ok P (2012). Bacteria-reactive immune response may induce RANKL-expressing T cells in the mouse periapical bone loss lesion. J Endod.

[49] Baral P, Umans BD, Li L, Wallrapp A, Bist M, Kirschbaum T (2018). Nociceptor sensory neurons suppress neutrophil and gammadelta T cell responses in bacterial lung infections and lethal pneumonia. Nat Med.

[50] Boyle WJ, Simonet WS, Lacey DL (2003). Osteoclast differentiation and activation. Nature.

[51] Hofbauer LC, Khosla S, Dunstan CR, Lacey DL, Boyle WJ, Riggs BL (2000). The roles of osteoprotegerin and osteoprotegerin ligand in the paracrine regulation of bone resorption. J Bone Miner Res.

[52] Hou GQ, Guo C, Song GH, Fang N, Fan WJ, Chen XD (2013). Lipopolysaccharide (LPS) promotes osteoclast differentiation and activation by enhancing the MAPK pathway and COX-2 expression in RAW264.7 cells. Int J Mol Med.

[53] Martinho FC, Gomes BP (2008). Quantification of endotoxins and cultivable bacteria in root canal infection before and after chemomechanical preparation with 2.5% sodium hypochlorite. J Endod.

[54] He H, Chai J, Zhang S, Ding L, Yan P, Du W (2016). CGRP may regulate bone metabolism through stimulating osteoblast differentiation and inhibiting osteoclast formation. Mol Med Rep.

[55] Brazill JM, Beeve AT, Craft CS, Ivanusic JJ, Scheller EL (2019). Nerves in bone: evolving concepts in pain and anabolism. J Bone Miner Res.

[56] Chartier SR, Thompson ML, Longo G, Fealk MN, Majuta LA, Mantyh PW (2014). Exuberant sprouting of sensory and sympathetic nerve fibers in nonhealed bone fractures and the generation and maintenance of chronic skeletal pain. Pain.

[57] Novakovic J, Mardesic-Brakus S, Vukojevic K, Saraga-Babic M (2011). Developmental patterns of Ki-67, bcl-2 and caspase-3 proteins expression in the human upper jaw. Acta Histochem.

[58] Woo SM, Rosser J, Dusevich V, Kalajzic I, Bonewald LF (2011). Cell line IDG-SW3 replicates osteoblast-to-late-osteocyte differentiation in vitro and accelerates bone formation in vivo. J Bone Miner Res.

[59] Ecarot B, Desbarats M (1999). 1,25-(OH)2D3 down-regulates expression of Phex, a marker of the mature osteoblast. Endocrinology.

[60] Wang D, Christensen K, Chawla K, Xiao G, Krebsbach PH, Franceschi RT (1999). Isolation and characterization of MC3T3-E1 preosteoblast subclones with distinct in vitro and in vivo differentiation/mineralization potential. J Bone Miner Res.

[61] Yazid MD, Ariffin SHZ, Senafi S, Razak MA, Wahab RMA (2010). Determination of the differentiation capacities of murines’ primary mononucleated cells and MC3T3-E1 cells. Cancer Cell Int.

