# Supplementary figures and images for: Trigeminal neurons control immune-bone cell interaction and metabolism in apical periodontitis
Source: Cell Mol Life Sci. 2022 May 31;79(6):330. doi: 10.1007/s00018-022-04335-w (PMC9156470; doi:10.1007/s00018-022-04335-w)

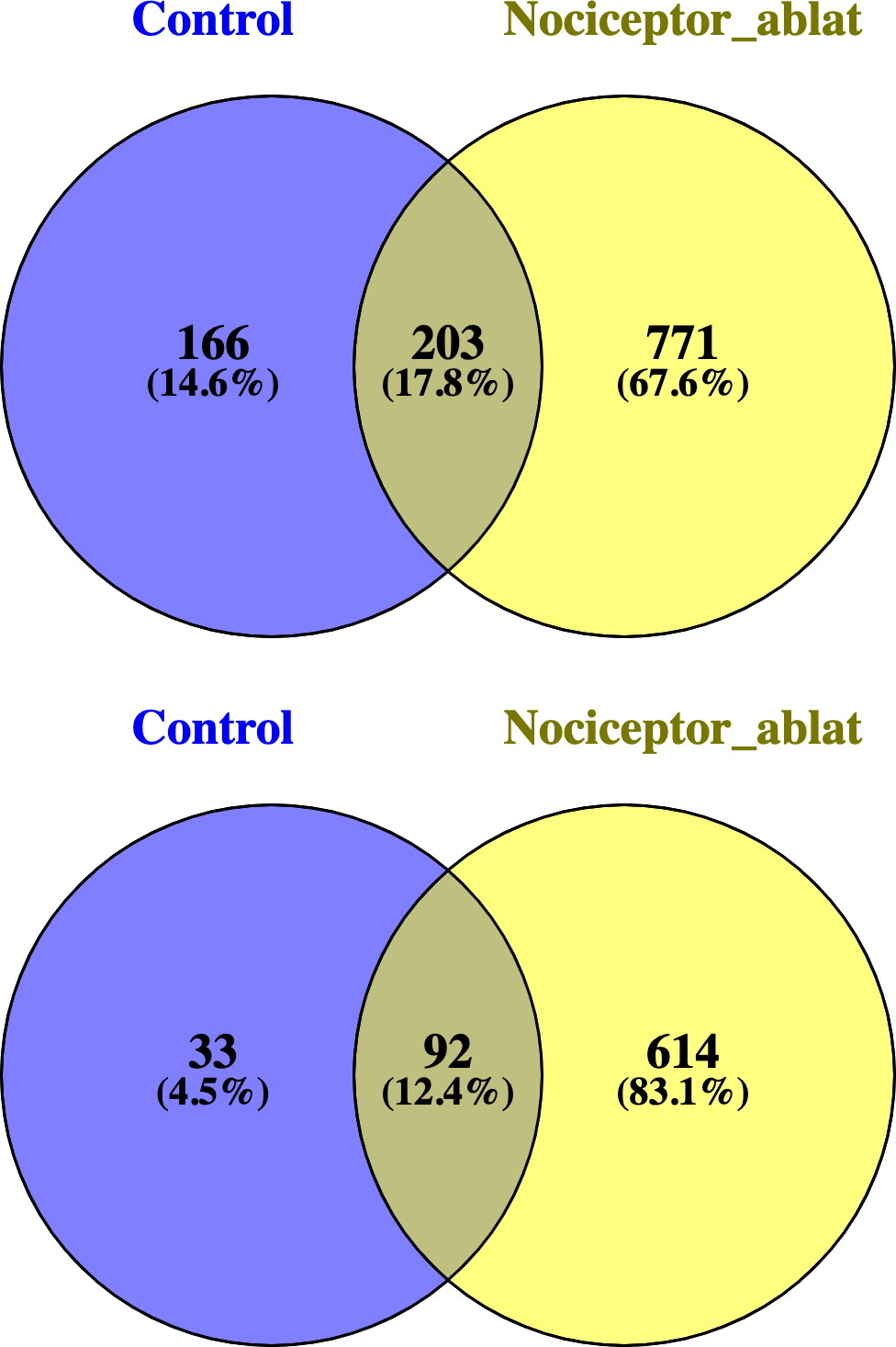

Supplement: Supplementary file 1 — Supplemental Figure 1. Nociceptors selectively regulate differential expression of genes in osteoblast precursors.Venn diagrams of distinct and common differentially expressed genes (FC>1.5, padj<0.05) for upregulated (1a) and downregulated (1b) genes in IDG-SW3 cells cultured with either Nav1.8-Cre or Nav1.8-DTA TG neurons. (PNG 119 KB) [file 18_2022_4335_MOESM1_ESM.png]
