# Supplementary material for: Trigeminal neurons control immune-bone cell interaction and metabolism in apical periodontitis
Source: Cell Mol Life Sci. 2022 May 31;79(6):330. doi: 10.1007/s00018-022-04335-w (PMC9156470; doi:10.1007/s00018-022-04335-w)
Supplement: Supplementary file 2 — Supplementary file2 (DOCX 14 KB) [file 18_2022_4335_MOESM2_ESM.docx]

Table 1. Summary of animals used

| **Mouse model** | **Source and Catalog #** | **Strain** | **Providing Laboratory** |
| --- | --- | --- | --- |
| Nav1.8 Cre | N/A | C57BL/6J | John Wood (University College London, UK) |
| Rosa26tm1(DTA)Lky/+ | Jackson Labs, 009669 | B6.129P2 | N/A |
