# Supplementary material for: Trigeminal neurons control immune-bone cell interaction and metabolism in apical periodontitis
Source: Cell Mol Life Sci. 2022 May 31;79(6):330. doi: 10.1007/s00018-022-04335-w (PMC9156470; doi:10.1007/s00018-022-04335-w)
Supplement: Supplementary file 3 — Supplementary file3 (DOCX 14 KB) [file 18_2022_4335_MOESM3_ESM.docx]

Table 2. Summary of genotyping primers used

| **Primer** | **Sequence 5’ →** **3’** |
| --- | --- |
| Nav1.8Cre, common forward | GGA ATG GGA TGG AGC TTC TTA C |
| Nav1.8Cre, wild type reverse | TTA CCC GGT GTG TGC TGT AGA AAG |
| Nav1.8Cre, mutant reverse | CAA ATG TTG CTG GAT AGT TTT TAC TGC C |
| DTA reporter, wild type forward | CCA AAG TCG CTC TGA GTT GTT ATC |
| DTA reporter, wild type reverse | GAG CGG GAG AAA TGG ATA TG |
| DTA reporter, mutant forward | CGA CCT GCA GGT CCT CG |
| DTA reporter, mutant reverse | CTC GAG TTT GTC CAA TTA TGT CAC |
