# Supplementary material for: Trigeminal neurons control immune-bone cell interaction and metabolism in apical periodontitis
Source: Cell Mol Life Sci. 2022 May 31;79(6):330. doi: 10.1007/s00018-022-04335-w (PMC9156470; doi:10.1007/s00018-022-04335-w)
Supplement: Supplementary file 4 — Supplementary file4 (DOCX 15 KB) [file 18_2022_4335_MOESM4_ESM.docx]

Table 3. Summary of immunohistochemistry reagents used

| **Epitope/Antigen or Product name** | **Source and Catalog #** | **Host species** | **Application** | **Dilution** | **Application specific details** |
| --- | --- | --- | --- | --- | --- |
| CD3 | Biolegends, #100324 | Hamster | IHC | 1:200 | Maxpack Immunostaining Media Kit |
| CD68 | Lifespan Bioscience, #LS-C188202 | Rat | IHC | 1:500 | Maxpack Immunostaining Media Kit |
| CGRP | Sigma-Aldrich, #C8198 | Rabbit | IHC | 1:300 | Maxpack Immunostaining Media Kit |
| Osteoprotegerin (OPG) | Abcam, ab73400 | Rabbit | IHC | 1:100 | Maxpack Immunostaining Media Kit |
| Receptor activator of nuclear factor κB ligand (RANKL) | Abcam, ab45039 | Mouse | IHC | 1:150 | Maxpack Immunostaining Media Kit |
| Ki67 | Novus, NBP3-05538 | Chicken | IHC | 1:1000 | Maxpack Immunostaining Media Kit |
| Osteocalcin | Takara Bio, M173 | Rabbit | IHC | 1:100 | Maxpack Immunostaining Media Kit |
| anti-Rabbit IgG (H+L) Cross-Adsorbed Secondary Antibody, Alexa Fluor 568 | Molecular Probes, A-11011 | Goat | IHC | 1:300 | Maxpack Immunostaining Media Kit |
| anti-Mouse IgG (H+L) Cross-Adsorbed Secondary Antibody, Alexa Fluor 488 | Molecular Probes, A-11001 | Goat | IHC | 1:300 | Maxpack Immunostaining Media Kit |
| VECTASHIELD® Antifade Mounting Medium with DAPI | Vector, H-1200-10 | N/A | IHC | N/A | 1 drop per slide |
