# Supplementary material for: Trigeminal neurons control immune-bone cell interaction and metabolism in apical periodontitis
Source: Cell Mol Life Sci. 2022 May 31;79(6):330. doi: 10.1007/s00018-022-04335-w (PMC9156470; doi:10.1007/s00018-022-04335-w)
Supplement: Supplementary file 5 — Supplementary file5 (DOCX 14 KB) [file 18_2022_4335_MOESM5_ESM.docx]

Table 4. Summary of cell lines used

| **Cell line** | **Source and Catalog# or RRID** | **Providing Laboratory** | **Species, cell type** |
| --- | --- | --- | --- |
| MC3T3-E1 Subclone 4 | ATCC, CRL-2593 | NA | Mouse calvaria osteoblasts |
| RAW 264.7 | ATCC, TIB-71 | NA | Mouse macrophage |
| IDG-SW3 | Kerafast, EKC001 | NA | Mouse long bone osteoblasts |
