# Supplementary material for: Trigeminal neurons control immune-bone cell interaction and metabolism in apical periodontitis
Source: Cell Mol Life Sci. 2022 May 31;79(6):330. doi: 10.1007/s00018-022-04335-w (PMC9156470; doi:10.1007/s00018-022-04335-w)
Supplement: Supplementary file 6 — Supplementary file6 (DOCX 14 KB) [file 18_2022_4335_MOESM6_ESM.docx]

Table 5. Summary of biological modulators used

| **Modulator (protein, small molecule)** | **Source, Catalog #** | **Solvent/ Vehicle** | **Concentration(s)** |
| --- | --- | --- | --- |
| LPS-EB Ultrapure | InvivoGen, tlrl-3pelps | 1X PBS | 100 ng/ml |
| RANK Ligand from mouse | Sigma, R0525 | 1X PBS | 100 ng/ml |
| cytosine β-D- arabinofuranoside (Ara-C) | Sigma, C1768 | media | 10μM |
